# The Evaluation and Comparing of Cytotoxic Effects of *Ferula gummosa Gum, Scutellaria lindbergii, Kelussia odoratissima and Artemisia kopetdaghensis* Extracts on ACHN Cell Line

**Published:** 2017

**Authors:** Azar Hosseini, Elham Bakhtiari, Abolfazl Khajavi Rad, Samira Shahraki, Seyed Hadi Mousavi, Shahrzad Havakhah, Mohammad Sadegh Amiri

**Affiliations:** a *Pharmacological Research Center of Medicinal Plants, School of Medicine, Mashhad University of Medical Sciences, Mashhad, Iran. *; b *Eye Research Center, School of Medicine, Mashhad University of Medical Sciences, Mashhad, Iran.*; c *Clinical Research Development Unit, School of Medicine, Mashhad University of Medical Sciences, Mashhad, Iran. *; d *Department of Physiology, School of Medicine, Mashhad University of Medical Sciences, Mashhad, Iran. *; e *Department of Biology, Payeme Noor University, Tehran, Iran.*; f *Medical Toxicology Research Center, School of Medicine, Mashhad University of Medical Sciences, Mashhad, Iran.*

**Keywords:** Apoptosis, Cytotoxicity, Ferula gummosa gum, *Scutellaria lindbergii*, *Kelussia odoratissima*, *Artemisia kopetdaghensis*

## Abstract

Renal cell carcinoma (RCC) is one of most fatal cancers. In most patients it is resistant to chemotherapy. *Ferula gummosa gum, Scutellaria lindbergii, Kelussia odoratissima, *and *Artemisia kopetdaghensis *are herbs about which there are some cytotoxic activity reports. In this study, cytotoxic and apoptotic activity of these four extracts on RCC cell line (ACHN) were evaluated and compared (ACHN) cells were treated with different concentrations of herbal extracts (15-500 μg/mL). Cell proliferation was determined after 24, 48, and 72 h. by MTT assay. Apoptotic cells were determined using PI staining of DNA fragmentation by ﬂow cytometry. Cell viability decreased with all herbal extracts in ACHN cells by 24, 48, and 72 h. as compared with control. Extracts induced a sub-G1 peak in ﬂow cytometry histogram of treated cells indicating apoptotic cell death is involved in extracts induced-toxicity. Results imply that four herbal extracts inhibit the growth of ACHN cells as a concentration- and time-dependent manner. Also, results show that apoptosis is proposed as the possible mechanism of action. So, four herbal extracts could be considered as good anticancer agents in RCC after further studies.

## Introduction

Renal cancer accounts for nearly 2% of all cancers generally. The American Cancer Society expected about 36,160 new cases of kidney cancer in 2005. Over 80% of kidney cancers are renal cell carcinomas (RCC), and the rest are mainly renal pelvis cancers. Incidence ratio of death in RCC is higher compared with other urologic cancers. RCC is unpredictable even when diagnosed and treated early by nephrectomy. The neoplasm can remain constant for years and then metastasis to other body organs (1). RCC is considered by absence of specific clinical signs. So it doesn’t allow the diagnosis at an early stage (2). Therefore high percentage of patients will have metastasis at the first diagnosis and can’t be cured. Unfortunately, there is no effective treatment for metastatic renal cancer. Classic approaches to RCC, such as radiotherapy, chemotherapy, or hormone- therapy have little or even no effect on this cancer (2). Immune-modulating agents, cytokines and differentiating agents, including retinoids, have shown antitumor activity in a low percentage of patients with metastatic RCC (3-5). These approaches don’t trace metastasis and even can stimulate tumor development by impairing the immune system. So, investigation of new approaches which focused on the regulation of tumor proliferation to effectively control RCC is necessary. Progress in the treatment of RCC has been little in the past 30 years and no effective chemotherapeutic agent presently is available against its (6, 7). So, there is a need for new and more selective agents with ability to affect targets directly involved in the progression of RCC development. On the other hand, the biological heterogeneity of RCC, its resistance to chemotherapeutic agents, and the several side effects of chemotherapeutics are the major problems in the treatment of RCC. The rate of systemic metastasis in RCC is high with approximately 50% of the patients with developing metastasis after surgical resection. So, radical nephrectomy of localized RCC is effective only in a few patients (8, 9). Therefore, the investigation for effective therapeutic agents for this cancer is urgently needed. Antioxidant-rich foods have many defensive properties against different diseases including neurologic degeneration, inflammatory disorders, coronary disease, and cancer (10, 11). *Scutellaria *L*.* (Lamiaceae) is a genus that contains about 300 species in the world, excluding South Africa (12). *Scutellaria *L*.* has 27 species and two hybrids in Iran. Among these 10 species and two hybrids are endemic to the country (13, 14). *Scutellaria lindbergii* Rech.f. is an Iranian species of this genus. Common distribution of this species is limited to Iran and Afghanistan (14, 15). In folk medicine, *Scutellaria *L*.*as a flavonoid rich plant is used for treatment of several diseases. Many studies have shown various biological and pharmacological activities of some Scutellaria species including antibacterial (16-18), antiviral (19), antifungal (20), anti-inflammatory (21), antioxidant (22), cytotoxicity, and anticancer (23-25). Several researches have been carried out on some biological effects of *Scutellaria lindbergii* including its antimicrobial, antioxidant (26) and cytotoxic activities (24). The genus *Ferula* belongs to the family Apiaceae and has about 170 species. These are produced from central Asia westward to northern Africa (27). The Iranian flora includes 30 species of Ferula that some of them are endemic (28, 29). The common Persian name of the most species is “Koma”. Ferula have been explored chemically (30-32). The members of this genus are well known as a good source of biologically active compounds such as derivatives (33-35), and sulfur containing compounds (36-38). *F. gummosa* was used astonic, anti-convulsant, anti-hysteric, decongestant and it is useful in treatment of neurological disorders, and stomachache (39-42). Umbelliferae comprises more than 450 genera and near 3700 species in the world (43). Kelussia is one of the most new genus of this family and is represented by only one species, *Kelussia odoratissima Mozaff. *which is found only in Iran (44). This sweet-smelling, self-growing monotypic medicinal plant is endemic to a limited area in western of Iran and is popularly called Karafse-Koohi. The aerial parts of the plant are usually used as a popular garnish and sedative. In traditional medicine, *K. odoratissima* is consumed to treat hypertension, cardiovascular diseases, and inflammation ulcers (45). The antioxidant properties of the methanolic extract of the plant were investigated by several methods.* Artemisia kopetdaghensis*, aromatic shrubs belonging to the Asteraceae family, is traditionally used in Iran as anti-inflammatory, antimicrobial, antifungal, and sedative (46, 47). In this study cytotoxic and apoptotic effects of different extracts were examined and compared on ACHN malignant cell line.

## Experimental

*Cell line and agents* **: **Renal carcinoma cell line, ACHN, was obtained from the Pasteur Institute, Tehran, Iran. 4, 5-Dimethylthiazol-2-yl, 2, 5-diphenyl tetrazolium (MTT) and Dulbecco’s Phosphate-buffered saline (PBS) were purchased from Sigma (St Louis, MO, USA). Propidium iodide (PI), sodium citrate, and Triton X-100 were purchased from Sigma (St. Louis, MO, USA). Fetal bovine serum (FBS), Glucose-high Dulbecco’s modified Eagle’s medium (DMEM), and penicillin streptomycin were purchased from Gibco (Grand Island, NY). Dimethyl sulfoxide (DMSO) was purchased from Merck.

**Figure 1:**
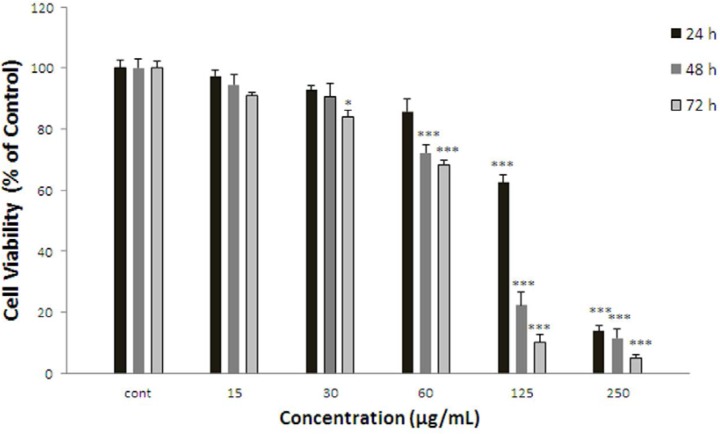
Effect of F.gummosa gum on cell viability of ACHN. Cells were treated with different concentrations of extract for 24, 48 and 72 h. Viability were quantitated by MTT assay. Results are mean ± SEM (n = 3). The percentage cell viability was normalized against the control. *** *P*<0.001, * *P*<0.05

**Figure 2:**
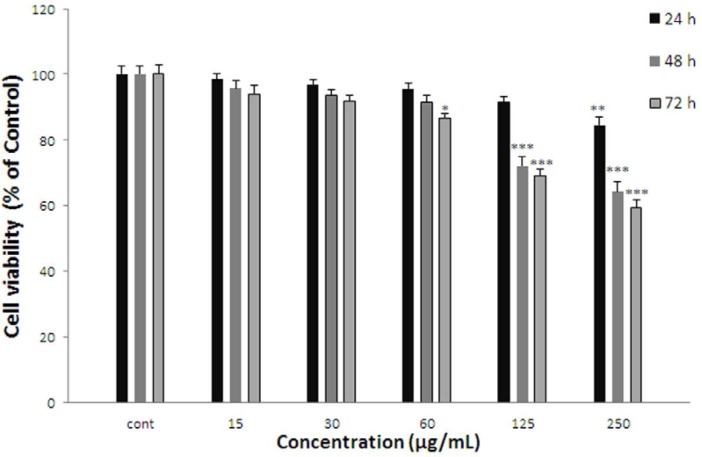
Effect of *A. kopetdaghensis* on cell viability of ACHN. Cells were treated with different concentrations of extract for 24, 48 and 72 h. Viability were quantitated by MTT assay. Results are mean ± SEM (n = 3). The percentage cell viability was normalized against the control. *** *P*<0.001, ** *P*<0.01, * *P*<0.05

**Figure 3:**
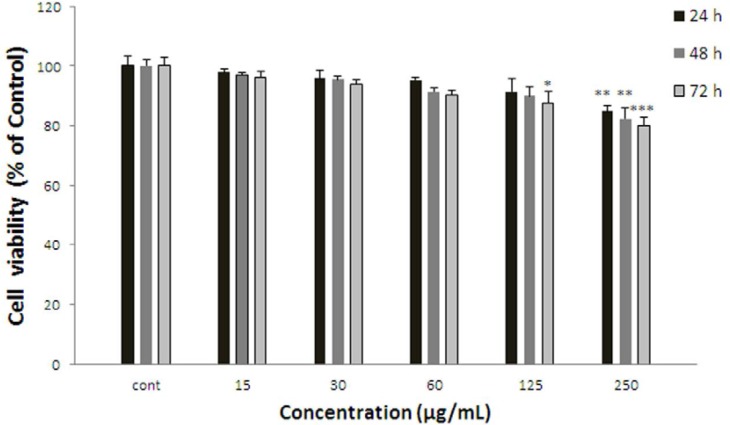
Effect of *S. lindbergii* on cell viability of ACHN. Cells were treated with different concentrations of extract for 24, 48 and 72 h. Viability were quantitated by MTT assay. Results are mean ± SEM (n = 3). The percentage cell viability was normalized against the control. *** *P*<0.001, ** *P*<0.01, * *P*<0.05

**Figure 4:**
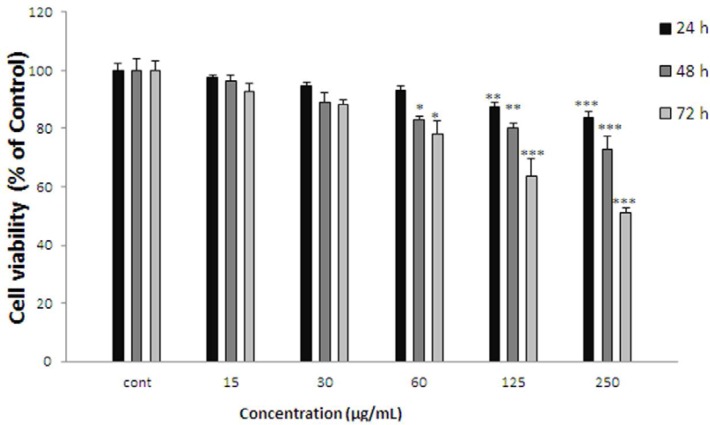
Effect of *K.odoratissima* on cell viability of ACHN. Cells were treated with different concentrations of extract for 24, 48 and 72 h. Viability were quantitated by MTT assay. Results are mean ± SEM (n = 3). The percentage cell viability was normalized against the control. *** *P*<0.001, ** *P*<0.01, * *P*<0.05

**Figure 5:**
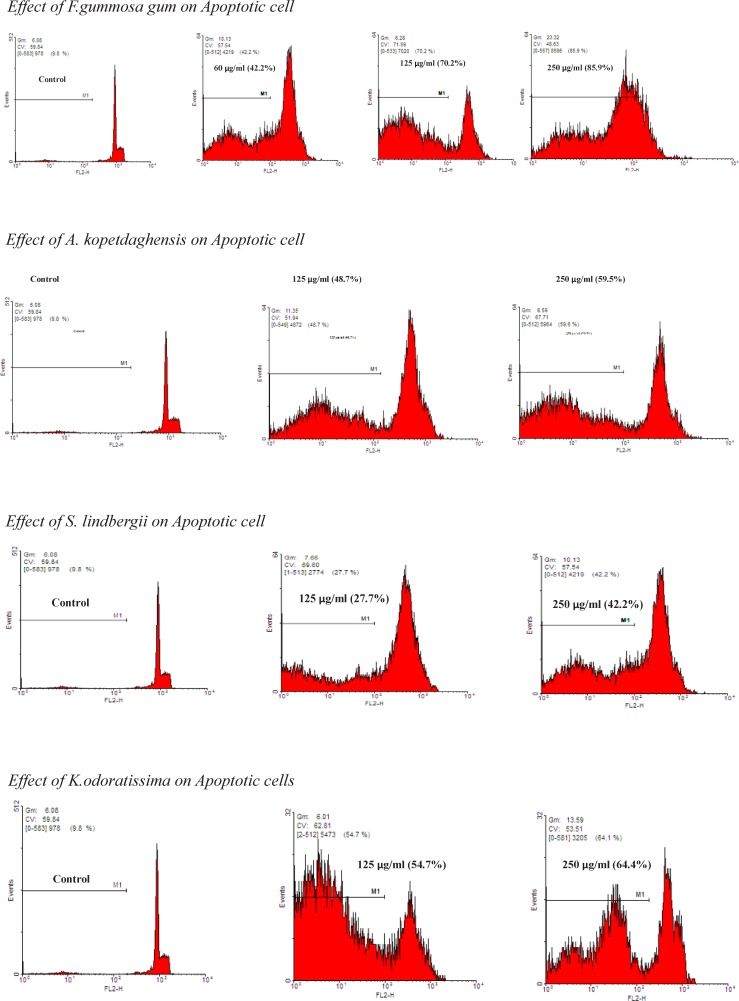
The proportion of apoptotic cells was measured with PI staining of DNA fragmentation by flow cytometry. The extracts induced a sub-G1peak (one of the reliable biochemical markers of apoptosis) in flow cytometry histogram of treated cells compared to control

*Cell culture*

ACHN Cells were maintained at 37 °C in a humidified atmosphere containing 5% CO2. The cells were cultured in DMEM supplemented with 10% fetal bovine serum, 100 Units/mL penicillin and 100 µg/mL streptomycin. All cells were maintained in a humidified atmosphere (90%) containing 5% CO_2_ at 37 °C.

*Preparation of different extracts*

Aerial parts of *K. odoratissima* were collected from Zard-Kooh Mountains, Charmahal-e-Bakhtiari; *F.gummosa* gum was collected from the roots of *F.gummosa*. The fresh *A. kopetdaghensis* was collected from Gonabad (Eastern area of Iran) and identified by the herbarium of Ferdowsi University of Mashhad, Iran (voucher specimen number: 35205). *S.lindbergii* was collected from Kang valley (height1800 m) near Mashhad (Razavi Khorasan Province, northeast of Iran) in July (2014) and identified by Mr. M. R. Joharchi, from Ferdowsi University of Mashhad Herbarium (FUMH).A voucher specimen of the species was deposited in the Herbarium of the Faculty of Pharmacy, Mashhad University of Medical Sciences (MUMS) under number: 11309.The herbs were dried, powdered, and subjected to extraction with 70% ethanol in a Sox let apparatus for 48 hr. The extracts were then dried on a water bath and dissolved in DMSO.

*Cell proliferation (MTT) assay*

Cells (5000/well) were seeded out in 96-well culture plate and after 24 h. he cells were treated with different extracts and then incubated for another 24, 48, and 72 h. MTT solution in phosphate-buffered saline (5 mg/mL) was added to each well at final concentration of 0.05%. After 3 h. the formazan precipitate was dissolved in DMSO. The absorbance at 570 and 620 nm (background) was measured using a Stat FAX303 plate reader. All treatments were carried out in triplicate.

*PI Staining*

Apoptotic cells were detected using PI staining of small DNA fragments followed by flow cytometry. It has been described that a sub-G1 peak that is reflective of DNA fragmentation can be observed following the incubation of cells in a hypotonic phosphate-citrate buffer containing a quantitative DNA-binding dye, such as PI. Apoptotic cells that have lost DNA will take up less stain and appear on the left side of the G1 peak in the histogram. Briefly, ACHN cells were seeded in wells of a 24-well plate overnight. Then, the cells were treated with different concentrations of extracts for 24 h. Floating and adherent cells were then harvested and incubated at 4 °C overnight in the dark with 750 µL of a hypotonic buffer (50 µg/mL PI in 0.1% sodium citrate with 0.1% Triton X-100). Next, flow cytometry was carried out using a FACS can flow cytometer (Becton Dickinson). A total of 10000 events were acquired with FACS.

*Statistics*

All results were expressed as mean ± SEM. Statistical analysis was performed using one way analysis of variance (ANOVA) followed by Bonferroni’s post-hoc test. Differences were considered significant at p < 0.05.

## Results

*Effect of F.gummosa gum on ACHN cell viability*

ACHN cells were incubated with different concentrations of *F. gummosa* gum extract (15-250 µg/mL) for 24, 48, and 72 h. As shown in Figure 1, after 24 h. iability of ACHN, cells as a concentration dependent manner decreased with *F. gummosa* gum extract to 62.62±2.55% and 13.48±1.74% for concentrations of 125 and 250 µg/mL respectively (*P* < 0.001 at concentrations of 125-250 µg/mL). After 48 h. cell viability decreased to 72.16 ± 2.82%, 22.37±4.09% and 11.53±3.02% for concentrations of 60-250 µg/mL respectively (*P* < 0.001 at concentrations of 60-250 µg/mL). Also, after 72 h. cell viability with *F. gummosa* gum extract decreased to 83.93±2.16, 68.28±1.46, 10.08±2.77 and 5.02±0.98 at concentrations of 30-250 µg/mL respectively (*P* < 0.001 at concentrations of 60-250 µg/mL and* P* < 0.05 at concentration of 30 µg/mL).

*Effect of A. kopetdaghensis on cell viability*

ACHN cells were incubated with different concentrations of *A. kopetdaghensis* extract (15-250 µg/mL) for 24, 48, and 72 h. As shown in Figure 2. After 24 h. viability of ACHN cells as a concentration decreased with *A. kopetdaghensis* extract to 84.58±2.34% for 250 µg/mL of extract (*P* < 0.01 at concentration of 250 µg/mL). Cell viability after 48 h decreased to 82.51±1.48%, 75.54±2.3%, 72.02±2.9%, and 64.41±2.95% at concentrations of 30-250 µg/mL respectively (*P* < 0.001 at concentrations of 60-250 µg/mL,* P* < 0.01 at concentration of 30 µg/mL). Also, after 72 h cell viability with *A. kopetdaghensis* extract decreased to 84.71±2.83%, 78.05±1.91%, 69.24±1.75%, and 59.42±2.52% for concentrations of 15-250 µg/mL respectively (*P* < 0.001 at concentrations of 30-250 µg/mL,* P* < 0.05 at concentration of 15 µg/mL).

*Effect of S. lindbergii on cell viability*

ACHN cells were incubated with different concentrations of *S. lindbergii* extract (15-250 µg/mL) for 24, 48, and 72 h. As shown in Figure 3, after 24 h. viability of ACHN cells as a concentration dependent decreased with *S. lindbergii* extract to 84.83±1.83% at 250 µg/mL of extract (*P* < 0.01 at concentration of 250 µg/mL). After 48 h. cell viability decreased to 82.24±3.52 % for 250 µg/mL of extract (*P* < 0.01 at concentration of 250 µg/mL). Also, after 72 h cell viability with *S. lindbergii* extract decreased to 87.65±3.66% and 79.91±2.78% for concentrations of 125 and 250 µg/mL respectively (*P* < 0.001 at concentration of 250 µg/mL, *P* < 0.05 at concentration of 125 µg/mL).

*Effect of K.odoratissima on cell viability*

ACHN cells were incubated with different concentrations of *K. odoratissima* extract (15-250 µg/mL) for 24, 48, and 72 h. As shown in Figure 4. After 24 h. viability of ACHN cells as a concentration dependent manner decreased with *K. odoratissima* extract to 80.25±1.87% at 250 µg/mL of extract (*P* < 0.001 at concentration of 250 µg/mL). After 48 h. cell viability decreased to 84.96±0.87%, 83.77±1.38%, and 72.99±4.23% at concentrations of 60-250 µg/mL respectively (*P* < 0.001 at concentration of 250 µg/mL, *P* < 0.01 at concentration of 125 µg/mL,* P* < 0.05 at concentration of 60 µg/mL). Also after 72 h. cell viability with *K. odoratissima* extract decreased to 83.01±1.4%, 67.07±2.71%, and 51.3±1.46% at concentrations of 60-250 µg/mL respectively (*P* < 0.001 at concentrations of 125-250 µg/mL, *P* < 0.05 at concentration of 60 µg/mL).

## Discussion

In recent years, the use of natural products such as fruits, vegetables, and herbs has been particularly considered because of having ant oxidative compounds. It is believed that many of natural products have the potential to act as anticancer agents in human (48). Many patients use natural products as alternative therapies for cancer or other chronic conditions (49). Several studies have demonstrated that antioxidant compounds have positive effects against different diseases, such as cancer, coronary diseases, inflammatory disorders, neurologic degeneration, and aging (10, 11). Whereas oxidative stress play role in cancer disease, as result herbal medicine can be have positive effects in cancer disease. The anti-cancer properties of medicinal herbs are mediated through different mechanisms including altered carcinogen metabolism, induction of DNA repair systems, immune activation, and suppression of cell cycle progression/induction of apoptosis (50). Herbal phytochemicals are used as promising resources for anticancer remedies or adjuvant for chemotherapeutic drugs to elevate their efﬁciency and reduce their side effects (51). RCC is the most deadly type of cancers of the urinary tract. Its incidence among males is more than females. Cigarette smoking, obesity, and hypertension are important risk factors for RCC. Surgery, hormone therapy, immunotherapy and chemotherapy are used for treatment (52, 53). Nowadays, herbal medicines are considered because of cheap and available. In this research, we evaluated and compared four different extracts on cell viability of ACHN cells. Recent studies have shown that these medicinal herbs contain different levels of antioxidant compounds. *S.lindbergii* (Lamiaceae) is Iranian species of Scutellaria. Cytotoxic properties of total methanol extract of *S. lindbergii *and its fractions were investigated on some cancer cell lines (54). Anti-proliferative effect of *S. linbergii* extract is related to its flavonoids and phenolic compounds. Many studies have shown that flavonoids and phenolic compounds are potent scavengers of free radicals such as hydroxyl and superoxide radicals (55). However, *S. linbergii* might have potential for the prevention and treatment of diseases or conditions resulting from oxidative stress such as cancer and aging (56). *A.kopetdaghensis*, aromatic shrubs belong to the Asteraceae family. The recent studies have shown the anti-cancer effect of other species of *Artemisia,* such as *A. conformist* (57). and *A. absinthium* (58). The recent study has reported cytotoxic effect of *A. indica* against four cancer cell lines A-549, THP-1, Caco-2 and HEP-2 (59).* A. capillaris* and *A. herba-alba* have shown significant anti-proliferative activity against the human oral cancer and acute lymphoblastic leukaemia (CEM) cell lines respectively (60, 61). The cytotoxic effect of Artemisia is due to increase in the amount of Bax protein, formation of DNA fragments, and finally induce apoptotic pathway (57). The genus *Ferula* belongs to the family Apiaceae. Recent studies have shown that ferula has toxicity effect against MCF-7 cell line (62). Ethanolic extract of *Ferula *decreased cell viability in BHY cells (63). And induced apoptosis in gastric cancer cell line (64). Also, *F gummosa Boiss*. ﬂower and leaf extracts have high phenolic and ﬂavonoid contents. Therefore, anti-cancer effects observed by* F. gummosa Boiss* might be due to presence of the components (65).* Kelussia *is one of the newest genera of this family and is found only in Iran. The antioxidant activity of the methanolic extract of the plant was evaluated by several methods (45).Hence, the antioxidant activity of these herbs has been reported; as a result we evaluated the cytotoxicity effect of them on ACHN for the first time. Our findings showed that all extracts decreased cell viability as time and concentration dependently manner, but *Ferula gummosa* gum had more cytotoxicity and apoptotic activity in comparison with other extracts. 

## Conclusion

According to results of the present study,* Ferula* could be considered as source for natural cytotoxic compounds that can inhibit the proliferation of ACHN cells with involvement of apoptosis or programmed cell death. Further studies are needed to fully recognize the mechanism involved in cell death. Ferula could be considered as a promising agent in kidney cancer treatment.
